# The Amazing Evolutionary Complexity of Eukaryotic Tubulins: Lessons from *Naegleria* and the Multi-tubulin Hypothesis

**DOI:** 10.3389/fcell.2022.867374

**Published:** 2022-04-25

**Authors:** Chandler Fulton

**Affiliations:** Department of Biology, Brandeis University, Waltham, MA, United States

**Keywords:** tubulin isotypes, multi-tubulin, microtubules, *naegleria*, evolution, protists, heterolobosea

## Abstract

The multi-tubulin hypothesis proposed in 1976 was motivated by finding that the tubulin to build the flagellar apparatus was synthesized *de novo* during the optional differentiation of *Naegleria* from walking amoebae to swimming flagellates. In the next decade, with the tools of cloning and sequencing, we were able to establish that the rate of flagellar tubulin synthesis in *Naegleria* is determined by the abundance of flagellar *α*- and *β*-tubulin mRNAs. These experiments also established that the tubulins for *Naegleria* mitosis were encoded by separate, divergent genes, candidates for which remain incompletely characterized. Meanwhile an unanticipated abundance of tubulin isotypes has been discovered by other researchers. Together with the surprises of genome complexity, these tubulin isotypes require us to rethink how we might utilize the opportunities and challenges offered by the evolutionary diversity of eukaryotes.

## Introduction

The multi-tubulin hypothesis (Fulton and Simpson, 1976) was presented in a fully subscribed 5-day meeting on Cell Motility at Cold Spring Harbor Laboratory, which included 92 papers and was published in three volumes (Goldman et al., 1976). I presented our paper late one evening in the old Lecture Hall; the auditorium remained crowded despite the late hour. Talks were allowed 15 min. The audience was respectful, but our hypothesis was counter to the widely accepted, sensible hypothesis that tubulin was tubulin, and a pool of tubulin subunits were drawn upon to assemble microtubules for different uses, such as for mitosis and for assembling flagellar axonemes (references in Fulton and Simpson, 1976). After the talk I fielded some tough questions. One of my colleagues felt strongly enough to state that our hypothesis could not be true, we must be missing something. Her comment reflected the opinion of many. Such reactions certainly made me realize how strongly we were swimming against the current.

Here I summarize the results that led us to this hypothesis 45 years ago, based on our study of cell differentiation in the free-living, single-celled amoeboflagellate *Naegleria*, followed by what we have learned from our studies since then about multiple tubulin isotypes in this organism. This is followed by a discussion of what *Naegleria* and the multi-tubulins have taught us about the incredible diversity of eukaryotes, about how evolution has tinkered with basic building blocks of eukaryotes, and about what “far-out” organisms like *Naegleria* can contribute to biology.

## How *Naegleria* Led US to the Multi-Tubulin Hypothesis

I started my career as a faculty member in 1960. As a graduate student I had led two lives. I was fortunate to have been mentored in microbial genetics as the first student of Norton Zinder, who had recently discovered genetic transduction and “infective heredity” (Zinder, 1992). This was a time when microbial genetics was becoming molecular biology. I was particularly impressed by Jacob and Monod’s awesome achievement of discovering the pathways by which gene expression in *E. coli* and in temperate bacteriophage *λ* were regulated, something about which virtually nothing was previously known, entirely by using classical genetics of mutants and crosses (Jacob and Monod, 1961).

Simultaneously, I had become fascinated by the mystery of eukaryotic cell differentiation. My undergraduate education was rich in classical biology, including invertebrate biology, where I learned about how all eukaryotes had similar cells but used them to build different body plans (e.g., Buchsbaum, 1948). While a graduate student, I took and then for three summers taught in the Embryology Course at the Marine Biological Laboratory, under the direction of Mac V. Edds. When I started independent research, I wanted to combine these two interests: to induce eukaryotic cells to rapidly differentiate from one major phenotype (A) to another (B), and then use genetics to dissect the mechanism. I explored organisms from vertebrate cells to single-celled algae, and ultimately chose the amoeboflagellate *Naegleria*. I was familiar with this little-studied protist because I had encountered it as a contaminant in 1958 when I attempted to grow hydra cells in culture, in hopes of regenerating hydra from a cloned cell. The hydra cells never grew, but I was initially misled by what proved to be a contaminant that looked “just like” hydra cells, amoebae representing ectoderm, and flagellates endodermal cells. From protozoology books I learned the contaminant was a free-living protozoan called *Naegleria*. It taught me how easily one could imagine hydra as a multicellular amoeboflagellate.

**FIGURE 1:**
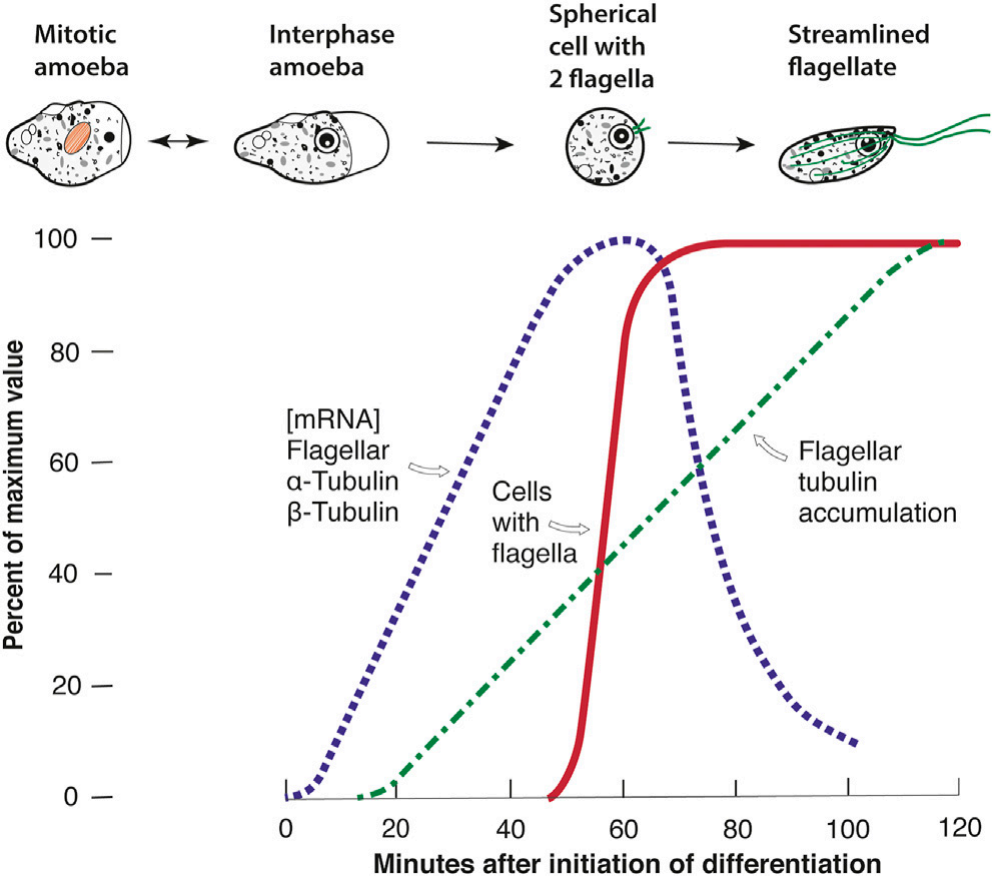
The tubulin repertoire of *Naegleria gruberi* NEG. Interphase amoebae lack any cytoplasmic (or nuclear) microtubules. Dividing amoebae assemble mitotic tubulins (shown in orange) during their intranuclear mitosis. During the optional differentiation from amoebae to flagellates, induced by transfer from growth environment to nutrient-free buffer, the cells undergo a dramatic phenotypic change to rapidly swimming flagellates. The cells lose their capacity for amoeboid movement and round up. Basal bodies form and move to the cell surface, where they nucleate the growth of flagella. As the flagella elongate so do the cells, forming a streamlined shape with a cytoplasmic cytoskeleton of microtubules (shown in green). The differentiation can be made synchronous and temporarily reproducible. The graph shows the one-step differentiation experiment, measured as a quantal change (the appearance of flagella on fixed cell samples), and quantitative changes in flagellar tubulin mRNA and protein. See text for references.

When I had my own lab and we began searching for a single-celled eukaryote that underwent a dramatic phenotypic change (A to B), the change of *Naegleria* from amoebae to flagellates (A–F) came to mind. We obtained some *Naegleria*, domesticated the organism, and trained it to undergo a rapid, synchronous, and temporally reproducible differentiation on command (Figure 1, red line) (Fulton and Dingle, 1967; Fulton, 1977a). The differentiation can be induced under the experimenter’s control simply by transfer of amoebae from a growth environment to a nutrient-free environment. It is completely optional, as the amoebae can grow for many hundreds of generations without ever differentiating. In the laboratory, the flagellates are transient, and eventually revert to amoebae. To this day this one-step differentiation remains one of the most controllable phenotypic changes available. Under the conditions used for the experiments in Figure 1, half the cells of *N. gruberi* NEG have visible flagella at 60 min (the T_50_). Anticipating genetics, we began to isolate an array of mutants (Fulton, 1970), but to this day there is no laboratory genetics for *Naegleria* [even though we know from more recent work that the cloned strain we mostly utilize, *N. gruberi* NEG, is a diploid that almost certainly arose from a mating before it was collected from nature 60 years ago (Fritz-Laylin et al., 2010)].

One dramatic feature of the quick-change act is the formation of flagella and of a streamlined flagellate body shape. Overall, *Naegleria* has a remarkable microtubule wardrobe. The amoebae have microtubules only in their mitotic spindle. Interphase amoebae have no microtubules in their cytoplasm or nucleus [many references, including (Walsh, 1984)]. The only commonly studied eukaryote I know that displays such an extreme absence of cytoplasmic microtubules is interphase amoebae of *Entamoeba* (Meza et al., 2006), which is a member of the Amoebozoa supergroup that diverged at the base of the animal cell lineage (Eichinger et al., 2005), and thus is much more closely related to animals than to the much earlier diverging Excavates, the diverse group to which *Naegleria* belongs (as described later).

**FIGURE 2:**
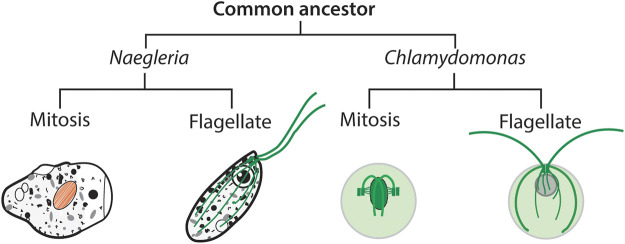
The last eukaryotic common ancestor (LECA) of *Naegleria* and *Chlamydomonas* separated about 2,000 million years ago (Hedges et al., 2004), by which time LECA used microtubules for eukaryotic mitosis and for 9 + 2 flagellar axonemes with 9-triplet basal bodies. In *Chlamydomonas*, a single *α*-tubulin and single *β*-tubulin suffice for the mitotic spindle, the basal bodies, the flagella, and various accessory cytoplasmic microtubules in both dividing and swimming cells. In *Naegleria*, the flagellates synthesize very conserved *α*- and *β*-tubulin subunits to build the flagellar apparatus (shown in green), and some highly divergent protein(s) to assemble the mitotic spindle (shown in orange). The *Chlamydomonas* schematic diagrams are based on (Cross and Umen, 2015).

*Naegleria* amoebae appear to utilize actin-based motility machines almost entirely, and shut down the synthesis of actin and other components promptly during differentiation. As far as I am aware, *Naegleria* (and a few very close relatives) are unique in this combination: a primarily actin-based motility machine switching to a primarily tubulin-based motility machine—a dramatic switch which justifies its being referred to as a “yin-yang organism” (Lai et al., 1984). During mitosis the cells assemble a “closed mitosis,” which means the nuclear envelope does not disassemble. Mitosis is also “acentriolar,” meaning the microtubules do not focus to cell centers. The mitosis is relatively barrel shape. This pattern of closed acentriolar mitosis seems unusual to those who focus on vertebrate cells, but is quite common among eukaryotes. Examples of organisms that use either closed or open mitoses are found in every major eukaryotic group except for the Excavates, in which only organisms with closed mitoses have been observed so far (Boettcher and Barral, 2013). An example of a closed mitosis is seen in a green alga in which the cell centers do not form the cell poles, as shown in Figure 2. Among the Myxomycetes in the Amoebozoa group, some individual species like *Physarum polycephalum* employ open mitosis with centrioles as cell centers in their single-celled amoeboid phase, but closed mitoses without centrioles in their syncytial “slime mold” phase (Fulton, 1970, p. 388; Solnica-Krezel et al., 1991). Thus even a single organism can alternate between these fundamental forms of mitosis.

In mitotic *Naegleria*, the microtubules appear quite normal at electron microscope resolution (Fulton, 1970, Figure 11; Schuster, 1975). Walsh has published a magisterial study of mitosis (Walsh, 2012), to which (Velle et al., 2022) offer some helpful additions. Nothing known about mitosis suggests that its tubulin would be “abnormal.” Unusually in *Naegleria* the nucleolus does not disassemble during mitosis, but divides with the chromosomes (Walsh, 2012). The mitosis is also efficient; remarkably these 15 µm diameter eukaryotic cells are able to divide as frequently as every 1.7 h (Fulton and Dingle, 1967). Mitosis, from prophase to cytokinesis, has been estimated to take 15–20 min (Fulton, 1970, p. 386).

The individual chromosomes are difficult to resolve in electron micrographs (Fulton, 1970; Schuster, 1975). The chromosomes are tiny, and too small to count, but a normal mitotic chromosome cycle can be visualized using Feulgen stain (Rafalko, 1947; Fulton, 1970), orcein stain (Fulton and Guerrini, 1969), or DAPI fluorescence (Walsh, 2012).

When these amoebae are induced to differentiate to flagellates, the cells turn their attention to tubulins. As differentiation progresses, these tubulins are assembled, successively, into the centriole-like basal bodies, which occurs about 10 min before flagella are visible. This feature of *Naegleria*, the *de novo* formation of centriole structures, also came as an unexpected surprise to the paradigm of the essential continuity of centrioles when it was described (Fulton and Dingle, 1971). Subsequently, as the flagella are assembled and elongate from the cell surface, the flagellar axonemes are assembled (Dingle and Fulton, 1966), and then an array of cytoskeletal microtubules is assembled as the flagellates elongate and become streamlined (Fulton, 1977b; Walsh, 1984). The structures of the 9-triplet centrioles, the 9 + 2 axonemes, and the microtubule cytoskeleton are all canonical. Once the whole array is assembled, flagellates can swim about a hundred times faster than the amoebae can walk (Fulton, 1977b). The flagellates ultimately revert to amoebae.

Our first look at microtubules came with a series of surprises: no microtubules in non-mitotic amoebae, no centrioles, no detection of chromosomes in electron microscopy of dividing cells, *de novo* assembly of centrioles. It was becoming clear that *Naegleria* was not a single-celled representative of a “typical” eukaryotic cell. Yet in its microtubules, and their behavior, there was no indication of any structural or functional abnormalities. *Naegleria* was asserting, loudly and clearly: “I am not a single-celled animal. I have different lessons to teach.” Because of our proto-zoa (“first-animals”) bias, it took us a while to hear her message.

We assumed, based on prevailing knowledge, that the tubulin involved in assembling mitotic microtubules would provide a pool to be used in the assembly of the flagellar apparatus. For example, Inoué’s idea of the dynamic equilibrium between a tubulin pool and the reversible assembly of mitotic apparatus (Inoué and Sato, 1967) was widely accepted. The flagella offered a promising place to begin seeking to understand the molecular biology and regulatory events of differentiation, and microtubules were their dominant feature, so we began to look at tubulins. This led us inexorably toward the multi-tubulin hypothesis.

In our first study of tubulins, Joel Kowit and I purified flagellar outer doublets, and from batches of 4.5 × 10^10^ cells (containing 4.2 g of total cell protein) we obtained a yield of 1.5 mg of tubulin that was 93% pure (Kowit and Fulton, 1974b). This tubulin showed similarity to other tubulins in molecular weight, the electrophoretic mobility of *α*- and *β*-subunits, and amino acid composition. It was injected into a rabbit, which yielded an antiserum with antibodies specific to the tubulin of *Naegleria* flagellates. We measured the amount of antigen in amoebae and flagellates, and found to our surprise that at least 97–98% of the “flagellar tubulin antigen” arises during differentiation. The simplest explanation of the dramatic rise is that this specific tubulin is synthesized *de novo* during differentiation, although a model involving post-translational modification could account for the results.

In extensive subsequent use, our polyclonal antibodies to flagellar outer doublet tubulin have reacted, *via* immunostaining, with *Naegleria* basal bodies, flagella and cytoskeletal microtubules, but not with *Naegleria* mitotic spindles, and not with tubulins of other tested species, including sea urchin tubulins and ciliate cilia. Subsequently Charles Walsh developed monoclonal antibodies to *Naegleria* flagellar *α*- and *β*-tubulins that also recognize both *Naegleria* mitotic and flagellar microtubules as well as the tubulins of other species (Walsh, 1984). These monoclonal antibodies have been widely used.

To determine whether flagellar tubulin was truly synthesized *de novo*, we subsequently labeled amoebae with ^35^S-methionine, and then during differentiation “chased” this radioactivity with as much unlabeled methionine as did not affect differentiation. These pulse-chase experiments showed that at least 70% of the flagellar tubulin was assembled *de novo* during differentiation, which is a minimum estimate since during growth some of the radioactive methionine was converted to cysteine and this ^35^S could not be chased during differentiation. Using both the pulse-chase experiments and our anti-flagellar tubulin antibody we were able to reveal the timetable of synthesis of flagellar tubulin as shown in Figure 1, green dashed line (Kowit and Fulton, 1974a). We also showed that more flagellar tubulin was synthesized than was needed for assembly of flagella. For example, if we arrested all protein synthesis when 40% of the antigen had accumulated, the flagella still grew to full length (Fulton and Walsh, 1980).

The multi-tubulin hypothesis (Fulton and Simpson, 1976) confronted the puzzle presented by the *de novo* synthesis of flagellar tubulin during differentiation, which was also supported by additional data. An examination of the literature showed no examples where interconversion of a tubulin pool between more than one microtubule structure had been established, e.g., mitotic ↔ flagellar microtubules, although it seemed reasonable that this sometimes happened. The literature also provided examples, especially from studies of sea urchin tubulins by Ray Stephens and others (e.g., Fulton et al., 1971; Stephens, 1975), that could be more simply explained if more than one tubulin isotype were utilized.

The multi-tubulin hypothesis paper also discussed “tubulin pools” which were in vogue at the time, but subsequently we found that the estimated pool sizes were excessively large due to contamination of the samples with non-tubulin proteins (Fulton and Simpson, 1979). Fortunately, these estimates did not alter our argument.

At the time of this paper, the cloning and sequencing of genes had not been achieved, and we knew little about tubulin sequences in any organism, although with considerable effort some segments of sequence had been obtained (e.g., Luduena and Woodward, 1973). It was possible to explain away all the antibody results as due to changes like post-translational modifications, and even the *de novo* synthesis of *Naegleria* flagellar tubulin could be explained *in extremis* as the way this organism made sufficient tubulin during differentiation. A colleague could offer an interesting argument, as one did in a phone call to me, that our work “was not ready for prime time because we had not shown the precursor of flagellar tubulin,” something that would be impossible to do if the tubulin was, in fact, synthesized *de novo*. At the same time, we found no direct evidence in the literature that tubulin utilized to form one structure formed a pool that remained available to form another structure. Thus we were left with a conjecture that argued some microtubules used specific tubulin subunits made by specific genes. While tubulins within an organism did not appear to be entirely equal, it also seemed likely that common tubulin pools were repeatedly reutilized in a dynamic equilibrium with assembled microtubules, as in the successive mitoses during embryonic cleavages. The multi-tubulin hypothesis provided a stimulus for us and others to seek definite answers as soon as it became feasible.

## What We Have Learned About *NAEGLERIA*’S Tubulins since 1976

It was only a few years before biologists were able to clone and sequence genes, and to measure messenger RNA. Then the complexities of tubulins quickly became clear, and the multi-tubulin concept rapidly “evolved” to specific cases, which led to the ongoing excitement about tubulin isotypes.

First I focus on what we and others learned about *Naegleria*.

We were eager to measure the flagellar tubulin mRNA, but cloning and sequencing were not quite ready. The first major breakthrough came when Elaine Lai developed a procedure to measure flagellar tubulin mRNA by cell-free translation (Lai et al., 1979). The protocol involved three crucial steps: purify polyA-mRNA from successive time-points in synchronous differentiation, translate that mRNA in a cell-free system from wheat germ that was limited only by the amount of mRNA, and then measure the amount of translated flagellar tubulin produced using our antibody. The measurements produced the results shown in Figure 1, blue dotted line. Beginning early in differentiation, flagellar tubulin mRNA can first be detected, rises to a peak at about 60 min, and then declines with a half-life of 8 min. When these results were utilized to determine the rate of flagellar tubulin synthesis, the cumulative rate produced the curve of Figure 1, green dashed line, matching the previously measured accumulation of flagellar tubulin antigen. This revealed that the amount of flagellar tubulin antigen gave a true reflection of the synthesis and that the rate of synthesis was directly proportional to the amount of flagellar tubulin mRNA. This experiment also showed that there was no post-translational modification of flagellar tubulin specific to differentiating *Naegleria* that led to the reactivity of the flagellar tubulin with the antibody. Since we were interested in studying what induced flagellar tubulin gene expression during differentiation, we were very excited by these results. In separate experiments, we showed that both transcription and translation were essential for the differentiation and the synthesis of flagellar components, including tubulins (Fulton and Walsh, 1980).

Very shortly thereafter the cloning and sequencing of tubulin genes became possible, beginning with chicken tubulin genes (Cleveland et al., 1980). Soon there were families of tubulin genes. As new information became available, it quickly became clear that *α*- and *β*-tubulin subunits had diverged from a common ancestor, and that they had evolved to many similar but distinct isotypes that formed various heterodimers. The first description of a tissue-specific isotype came from Raff’s laboratory, the testis-specific *β*-2 tubulin isotype in *Drosophila* that is required for spermatogenesis (Kemphues et al., 1982).

*Naegleria* genes proved unexpectedly divergent, and in most cases it was challenging to clone their genes using heterologous probes from other organisms (e.g., chicken tubulin genes)—the highly efficient “cloning by phone” approach. Cloning the *Naegleria* genes took us several years—both because of the extensive separate evolution of these DNAs (see below) and because *Naegleria* genes are AT-rich (averaging 65% AT). We eventually cloned and sequenced flagellar *α*-tubulin genes (Lai et al., 1988) and *β*-tubulin genes (Lai et al., 1994). By focusing on differentiation-specific mRNAs, others cloned these genes in the related *N. pringsheimi* NB-1 (Mar et al., 1986; Shea and Walsh, 1987; Lee and Walsh, 1988). Our colleagues’ results and ours are fully concordant. The encoded tubulin sequences proved to be conserved, and the *Naegleria* flagellar tubulin monomers showed ≥90% similarity to those of many other organisms, from vertebrates to *Chlamydomonas*. Even though the encoded amino acid sequences were conserved, the DNA sequences had evolved so much as to make these invisible to heterologous probes (see discussion in Lai et al., 1988).

We cloned three *α*-tubulin and three *β*-tubulin genes, each representing three distinct genes but with only silent substitutions so they encoded identical subunits. We estimated the abundance of the genes by Southern blot analysis at about eight *α*-tubulins and eight to ten *β*-tubulins. In a separate study, an electrophoretic karyotype led us to estimate ∼23 chromosomes in *Naegleria*, and found most of the *α*-tubulin genes on one chromosome and the *β*-tubulin genes scattered on three or four chromosomes (Clark et al., 1990). In the genome project, a similar karyotype using the same strain led to an estimate of about 12 chromosomes (Fritz-Laylin et al., 2010). It is hoped someday that this information can be refined with genetic studies.

The cloning and sequencing of these genes revealed another unexpected result: in most organisms the *α*-tubulin encodes a C-terminal tyrosine, whereas in *Naegleria* and a few other species the C-terminal tyrosine is encoded on the *β*-tubulin subunit (Lai et al., 1994). These C-terminal tyrosines participate in a cycle of tyrosination and detyrosination, and it is of interest that this function can be served by a tyrosine at the terminus of either subunit. Other cases of exceptional tubulins have been described, such as a testis-specific chicken α-tubulin that lacks a terminal tyrosine (Pratt et al., 1987), and the tyrosination cycle has been extensively studied (Nieuwenhuis and Brummelkamp, 2019).

Using these *Naegleria* flagellar tubulin DNA clones, we could directly estimate the mRNA levels in amoebae and during differentiation. The results were striking: the abundance of both tubulin subunits rose and fell on exactly the timetable previously measured using translatable mRNA (Figure 1, blue-dotted line). The tubulin mRNAs become detectable quickly, within 10 min of differentiation (Lai et al., 1988). The abundance of translatable mRNA matched the abundance of physical, transcribed mRNA. In addition, no homologous mRNA could be detected prior to the onset of differentiation. This is shown by the absence of homologous *α*-tubulin mRNA even in heavily overloaded Northern blots, using RNA taken from exponentially growing amoebae (Figure 9 in Lai et al., 1988), as well as by seeking measurable *α*-tubulin mRNA in mitotically synchronized amoebae (synchronized as in Fulton and Guerrini, 1969). No trace of flagellar tubulin mRNA has been found except in differentiating cells. These experiments established that some other, divergent tubulin genes had to be responsible for the mitotic spindle, but the identity of these mitotic tubulin genes eluded us.

An exciting advance was made by Lee’s laboratory when they found an *α*-tubulin clone in a *Naegleria* closely related to strain *N. gruberi* NEG, now called *N. pringsheimi* strain NB-1 (Chung et al., 2002). This gene, which they called α6, encodes an *α*-tubulin of 452 amino acids that shows only 62% identity with *Naegleria* flagellar α-tubulin. They also found a partial clone of a similar but not identical gene, which was not further characterized. The encoded products of both genes were among the most exceptionally divergent of tubulin genes known. For example, the yeast *Saccharomyces cerevisiae* α-tubulin shows ≥70% identity to *Naegleria* or *Chlamydomonas* flagellar tubulin. The authors found that the *Naegleria* α6 gene was expressed in growing amoebae, but the expression was promptly shut down when differentiation was initiated. Finally, they prepared antibody to a peptide of the α6 sequence, and obtained faint staining of the nucleus of mitotic amoebae. Thus, to use their own words: “this report definitely proves the multi-tubulin hypothesis in *N. gruberi*.” Their excellent report left open one important question. The staining of the mitotic nucleus is light and not clearly localized to the microtubules. Again, to quote the authors: “it is not clear whether α6-tubulin is the only (or major) *α*-tubulin for mitotic spindle fiber microtubules or is a specialized α-tubulin that accounts for a minor percentage of the microtubules.” Determining whether such divergent tubulin as α6 is able, on its own, to build *Naegleria*’s mitotic spindle, remains an important question that needs to be answered using protein- and immuno-chemistry.

In 2010 the draft genome of *Naegleria gruberi* NEG-M (the axenic derivative of strain NEG) was completed (Fritz-Laylin et al., 2010). Among many surprises of *Naegleria*’s genome, which will be discussed shortly, this analysis permitted a survey of all the curated tubulins of this strain (presented as Supplementary Figure S4B in Fritz-Laylin et al., 2010). Redundant protein sequences were not counted, so the multiple *α*- and *β*-tubulin genes characterized previously—and estimated at eight to ten copies—are listed singly. They also reported a single *α*-tubulin gene with a sequence slightly divergent from the cloned sequence (“Naegrub 53,284”). They found strain NEG’s putative mitotic tubulin genes, a non-identical pair of α-tubulin genes similar to the α6 of strain NB-1, and in addition a pair of *β*-tubulin genes of similarly divergent nature which they deduced might be the mitotic *β*-tubulin genes. In addition, they reported a set of tubulins that I consider a “bonus,” highly divergent members of both families, with seven different *α*-tubulins and five *β*-tubulins. These are divergent not only in sequence, sharing at best about 60% encoded sequence with the flagellar tubulin genes, but also with gaps and additions which suggest they are unlikely to encode tubulins that would themselves assemble into microtubules. Finally, the genome was found to contain conserved, single, encoded gamma, epsilon and delta tubulins.

A preliminary paper extends the result of the Lee laboratory on strain NB-1 α6 tubulin (Velle et al., 2022). Both putative mitotic α-tubulin genes of strain *N. gruberi* NEG-M are shown to be expressed in growing amoebae, and this finding is extended to both putative mitotic *β*-tubulin genes of NEG-M, with none of the four genes expressed during differentiation. This finding supports the hypothesis that these divergent tubulin subunits, both *α* and *β*, play some role in growing amoebae, but still leaves open the question of whether they are either necessary or sufficient to build the mitotic microtubules. We need more direct evidence. These putative mitotic tubulin subunits are among the most divergent sequences found. I estimated a 61–64% identity of the two mitotic α to *Naegleria* flagellar α-tubulin subunits and 62–66% identity of the two mitotic *β* to *Naegleria* flagellar *β*-tubulin subunits, while most tubulins are conserved in the vicinity of ≥90% identity. None of these four putative mitotic *α*- or *β*-subunits encode a C-terminal tyrosine. It would almost be surprising if they can assemble into microtubules on their own. So while it remains clear that the mitotic spindles are built of something other than flagellar tubulin, we cannot conclude that these divergent tubulins are sufficient to build the mitotic microtubules until this question is answered by experiment. I suspect this investigation is likely to yield results of interest. Walsh has even raised the intriguing suggestion that perhaps a protein of the nucleolus, which co-divides with the chromosomes in *Naegleria*, may be involved in the spindle fibers (Walsh, 2012). Until biochemistry defines the structural components, we remain forced to the conclusion that the mitotic spindle is made of some tubulin(s) or proteins different from flagellar tubulin.

While this research with *Naegleria* was ongoing, many were obtaining fascinating results with tubulin isotypes in diverse organisms, from ciliates to *Drosophila*, yeast to mammals, including some protists [including a recent review focused on post-translational modifications in protists (Joachimiak and Wloga, 2021)]. Some results in other eukaryotic microorganisms make it clear that *Naegleria*’s use of very different tubulins for mitosis versus flagella is by no means the only evolutionary solution. Budding yeast, *Saccharomyces cerevisiae*, encode a single *β*-tubulin gene and they can be engineered to produce only one of two *α*-tubulin isotypes (Schatz et al., 1988; Luchniak et al., 2013). These yeast, with single *α*- and *β*-tubulin genes, appear to perform their microtubule functions, i.e., division, “normally.” But yeast do not make flagella.

Lest we allow the ability of some organisms, such as the exampled yeast, to manage with single tubulin genes, to dull our sensitivity to tubulin isotypes, an elegant new paper shows a surprise: that while budding yeast can be engineered to grow with single *α*- and *β*-tubulin genes, both α-tubulin genes in a wildtype yeast are functional in spindle positioning (Nsamba et al., 2021). As those studying tubulin isotypes have demonstrated repeatedly, there are subtleties in multiple tubulins. More await discovery.

The closely choreographed life cycle of the green alga *Chlamydomonas reinhardtii* uses microtubules for multiple functions (Johnson and Porter, 1968; Cross and Umen, 2015). Vegetative cells are flagellates that swim using two flagella, have classic 9-triplet basal bodies, and a complex array of microtubules forming a microtubular cytoskeleton. In anticipation of cell division, the basal bodies detach from the flagella, the latter then degenerate. The basal bodies each form a duplicate. Mitosis is closed, with an intact nuclear envelope, although some microtubules go through fenestrae in the envelope into the cytoplasm. Although the duplicated basal bodies are present through mitosis, the mitotic cells do not form apical poles focused on the centrioles, as in mammalian cells, but instead the two pairs of basal bodies associate with the cleavage furrow. Microtubules form the spindle and a complex of nuclear and cytoplasmic structures during division. As division into two cells is completed, the centrioles move to the cell membrane, and there new flagella are formed on each daughter cell. *Chlamydomonas* is haploid, with few excess genes (Merchant et al., 2007). It achieves its complex series of microtubule functions using duplicate *α*-tubulin and two *β*-tubulin genes (Youngblom et al., 1984; Silflow et al., 1985), which have been shown each to encode one identical heterodimer (Youngblom et al., 1984; James et al., 1993). Thus, as far as is known, this alga can accomplish its full repertoire of microtubular arrays using a single tubulin heterodimer, with whatever posttranslational modification and accessory proteins are involved in building the diverse structures. These four genes are turned on in tandem when regeneration of flagella is induced by excision (Silflow and Rosenbaum, 1981). Recently the *Chlamydomonas* tubulin genes have been disrupted using insertions that create null alleles (Kato-Minoura et al., 2020). This allowed the authors to engineer a *Chlamydomonas* mutant that had only one *α*-tubulin and one *β*-tubulin gene. This mutant grew at almost a normal rate and regenerated flagella normally after excision, revealing that “a single gene for each type is enough to supply the tubulin necessary for its cellular functions.” Thus it appears clear that *Chlamydomonas* can make a diversity of microtubules—singlets, doublets, and triplets—comparable to those found in *Naegleria* mitosis and flagellates, using a single *α*-*β*-tubulin heterodimer.

This presents us with a conundrum, illustrated in Figure 2. *Chlamydomonas* uses the same tubulin heterodimer for mitosis and flagella, whereas *Naegleria* goes to the trouble of having a separate set of flagellar tubulin genes and some sort of mitotic tubulin genes, whether or not these are the postulated putative mitotic tubulin genes. Aside from the conundrum of how this situation arose, the distinct mitotic and flagellar tubulins in *Naegleria* could provide modules for meeting the cells’ needs. In particular, when “time to differentiate” is signaled perhaps an army of about eight *α*- and eight *β*-tubulin genes need to be expressed simultaneously to produce sufficient flagellar tubulin on schedule (Figure 1). *Naegleria* amoebae respond to the command to differentiate promptly whether they are in early stationary phase—healthy but no longer preparing for another mitosis—or in log culture, or even if they are in the midst of preparing for synchronous cell division (Fulton and Guerrini, 1969). It seems clear that whatever amoebae are doing, if they have biosynthetic capacity when they receive the “differentiate” signal they drop everything and respond immediately (Fulton, 1977a, p. 614). This rapid response must have an important selective advantage, but despite more than a half-century of laboratory study we still do not know the role of the temporary flagellates in *Naegleria*’s worldwide success, so the advantage remains a mystery today. Yet, possessing a separate complementary set of tubulin genes might allow quick responses to “grow” vs. “differentiate” signals. What Moore and Wethekam have called “expression-control modules” may be a significant part of the advantages of multiple tubulins (Moore and Wethekam, 2021).

## What We Currently Know, and Don’t Know, About *Naegleria* Tubulins

Although we have learned a lot, important unanswered questions about *Naegleria* tubulins remain that merit investigation:

• While it is a reasonable inference that the highly divergent tubulin genes found in the *Naegleria* genome may be responsible in part or in full for the mitotic microtubules, this needs to be established by showing that the products of these genes are in fact assembled into the mitotic microtubules. Until then we may still have “surprises” ahead.
• If *Chlamydomonas*, for example, can use one set of tubulin genes for both tasks, and *Naegleria* and its relatives are virtually unique in possessing a divergent set of mitotic tubulin genes, how did this arise in evolution? As will be discussed further below, understanding the origin of *Naegleria*’s mitotic tubulins could be crucial to understanding early tubulin evolution.
• As to issues of multiple isotypes of tubulin, the finding of two sets of tubulin genes—flagellar and putative mitotic—in *Naegleria* does not give full understanding of the pattern of tubulin isotypes in *Naegleria*. There are multiple *α*- and *β*-tubulin genes in the flagellar sets, including some “bonus” subunits so divergent that while they are clearly tubulin subunits, they are not likely to assemble typical microtubules. Are these isotypes specific to basal bodies and their triplet fibers, or to specific components of the flagellar axoneme, or to the flagellate’s cytoskeleton?

Some of these experiments would require a gene editing method for knocking out individual genes, such as by using CRISPR-Cas. Up to this point *Naegleria* has successfully resisted any efforts to alter its genes by external manipulation. The efforts continue!

## What We Have Learned About the Unanticipated Diversity of Eukaryotes, and How This Diversity Can Contribute to Biology

Evolution!

No discovery in biology has produced so extended an argument as Darwin’s recognition of biological evolution. Everything about tubulins and their isotypes needs to be considered in the context of this ongoing excitement.

All eukaryotes depend on spindle microtubules for mitosis—loss of this function would apparently be a lethal mutation—and so great a diversity of eukaryotes possess canonical flagella and centrioles such that we can confidently assert that LECA, the Last Eucaryotic Common Ancestor, had both capabilities. It is clear that tubulin arose from prokaryotic proteins and developed as heterodimers of *α*- and *β*-subunits. All this was accomplished by LECA.

The growing ease of cloning and sequencing genes in the 1980’s, culminating with the first draft of the human genome by 2001, produced new data that led to a cataclysmic change in our understanding of eukaryotic diversity. This change rapidly altered the thinking of everyone concerned with eukaryotes and their genes (e.g., Lynch, 2007). These issues bring into focus several major aspects of our understanding of eukaryotes, and these in turn affect all of us interested in tubulin isotypes. One could explore each of these topics at length, but here I hope to raise a few of the most important considerations.

As Sydney Brenner so aptly put it: “We are all conscious today that we are drowning in a sea of data and starving for knowledge” (Brenner, 2002). As these data have accumulated, they have brought several realizations that undermined the precepts on which many based their approach to research in the 1960’s and 1970’s. The idea that eukaryotes were similar cells built on a basic plan that formed a diversity of cell types—from two or three types in a “first animal” like *Naegleria* to 200 or more in a mammal. This idea is expressed in a quotation attributed to Jacques Monod that “what’s true for *E. coli* is true for the elephant” (Monod and Jacob, 1961). [The origin of this famous statement is thought-provoking (Friedmann, 2004)]. In the 1960’s, the successes of genetics and biochemistry had lulled us into a sense that the unity of biology was such that we could define principles—like the *lac* operon or the Krebs cycle—and these would govern all eukaryotes. Ideas like these guided my early research career. Then came the tsunami of data. We were suddenly immersed in a total unanticipated diversity, of genes and of eukaryotes. We learned decisively that *Naegleria* is not a unicellular elephant!

Some might call “arrogant” the 1960’s attitude that using our brains we should be able to make sense of everything. Certainly evolution has nothing in its guiding forces that necessarily encourage it to build as an engineer would, or even to build in a manner that would make it easy for the human mind to grasp. Great messy complexities involving hundreds or thousands of interacting components can be selected for if they are functional, even if the contraption does not conform to a simple engineering diagram for us to understand. There is not any known mechanism guiding the forces driving evolution that specifies “if it ain’t broke don’t fix it” (an idea attributed to the 1970’s but one that presumably also guided stone age “engineers”). Even if something works, evolution has no hesitation to break it, and it is then left to selection to determine what happens next. If evolution destroys a mitotic apparatus, for example, that clearly is the end for that cell’s ability to reproduce.

When vertebrates and their cells, even human cells, became accessible to cloning and sequencing, the emphasis shifted from an interest in “all” eukaryotes as models to a narrow focus on our closer relations, and especially on mammals. This has been encouraged by the support mechanisms for science. Simultaneously, we are learning that the complexity of eukaryotes is far greater than anyone imagined in the 1960’s, or even a decade ago. And lest we become inclined to think that all we need to know will come from mammals and their ills, we should remind ourselves that about half the Nobel Prizes in Physiology and Medicine which have involved research that utilized organisms have gotten their insights based on research using non-vertebrate organisms, from bacteria (e.g., CRISPR) to yeasts (autophagy), from ciliates (telomeres) to flies (e.g., circadian rhythms).

It was early, and correctly, recognized that all known eukaryotes arose from a single ancestral cell, a last eukaryotic common ancestor (LECA), beginning perhaps as long ago as 2,200 mya (million years ago) or more (Parfrey et al., 2011), when oxygen was rising in the atmosphere.

There have been many attempts to reconstruct the phylogeny of eukaryotes from the LECA. At first ribosomal DNA sequences were used to build trees, and for a moment these trees looked like normal trees with a single main trunk (the common ancestor) branching off. *Naegleria* appeared to be an early-branching eukaryote (Hinkle and Sogin, 1993), but such trees between distant organisms were plagued by “long-branch attraction.” This method using ribosomal DNA sequences did lead correctly to the recognition that two groups previously considered very far apart, the animals and fungi, were in fact close relatives (Wainright et al., 1993).

By the time the *Naegleria* draft genome was completed (Fritz-Laylin et al., 2010), it had become clear that *Naegleria* was an early diverging eukaryote. By that time the immense diversity of eukaryotes had been loosely organized into five to eight “supergroups.”

An example of such a tree using supergroups is shown in the genome paper. The draft genome of the Heterolobosean amoeboflagellate *N. gruberi* NEG-M, the first of a free-living “excavate,” surprised us in many ways, including a large and complex gene repertoire, with over 15,727 curated genes that included abilities for anaerobic and aerobic respiration, and a surprising number of eukaryotic regulatory inventions for signaling, sexual, cytoskeletal, and metabolic modules (Fritz-Laylin et al., 2010).

After 2010, widespread sequencing of protist genomes has led to an explosion of new insights, results which make “the supergroup level even more arbitrary than before” and has reorganized dozens of early branches forming the eukaryotic tree (Burki et al., 2020). Such efforts are resolving the tree of life, and carry much new information, but the trees also are volatile and changing rapidly.

We focus here on a single group, the Heterolobosea, a part of the “excavate” subgroup (currently usually known as Discoba). As of 2014, and still today, the excavates—a world of little-known beasts with many fascinating properties—were the least studied of all supergroups in terms of genome sequences and of publications (Lynch et al., 2014). The Heterolobosea subgroup are a diverse group of mostly single-celled protists, of which *Naegleria* is the only “well known” member. The pioneering Heterolobosea separated from LECA very early, perhaps about 2000–2,100 mya (Hedges et al., 2004). At around the same time, their distant living relatives including *Euglena* and the trypanosomes (*Trypanosoma* and *Leishmania*) had begun their separate evolutionary journeys. When such generalizations are applied to all the groups of eukaryotic protists, the diversity is immense, as is our ignorance about them. Later separations from LECA include some of the best-known branches, including the one that led to plants and the one that led to the fungi-metazoa lineage.

Even within the genus *Naegleria*, there are roughly 40 species, and these include organisms that appear to be themselves quite diverse (De Jonckheere, 2004). For example, *N. gruberi* and the opportunistic human pathogen *N. fowleri* were long ago argued to be evolutionarily as distant from one another as are frogs and people (Baverstock et al., 1989), a striking conclusion about two members of a single genus. Remarkably this great evolutionary distance within the genus is supported by comparing large sets of their chromosomal genes using as an outgroup the distant trypanosomes, which lie outside the Heterolobosea (Liechti et al., 2019; their Figure 3)—although the authors of this exceptional tree make no comment about the extreme difference. Such evolutionary distances within a genus makes the diversity of protists even more exceptional.

So far the divergent putative mitotic tubulins have only been found within the few sequenced Heterolobosea (Velle et al., 2022). Now that the *N. fowleri* genome has been expanded (Liechti et al., 2019), when one searches databases using the putative mitotic tubulins from *N. gruberi*, one finds similar, but somewhat divergent, homologous pairs in the *N. fowleri* genome, as well as homologs to both flagellar tubulin subunits.

LECA had to assemble tubulins for a mitotic spindle and for a flagellar apparatus. Some organisms, such as *Chlamydomonas*, appear to utilize a common set *α*- and *β*-tubulins to assemble microtubules for these two functions. In a world of logic, this would imply that the common ancestor might have used its tubulin genes in this manner. The key question for *Naegleria* is: were separate tubulin isotypes originally used for mitosis and flagellar apparatus, or was an integrated set of specialized isotypes the “original” arrangement? If the original was a single set, then what event occurred that led to *Naegleria*’s having a set of flagellar tubulins and a set of very divergent mitotic tubulins? *Naegleria*’s system now works very well, allowing an amoeba to grow and divide rapidly. Indeed, for *Naegleria*, with only one cell type that reproduces, the fastest growing amoebae will “win” the evolutionary race at every generation, and the genus is common globally wherever there is fresh water. But how, from an engineer’s viewpoint, could this situation arise? At some point evolution had to switch over either from a single tubulin set that did both or from two specific sets. Tinkering with events as complex as the separation of a cell’s genes and the assembly of its flagellar apparatus is dangerous. Somehow we must invoke gene duplication and divergence, but this does not explain what happened. I think that when we understand how this occurred in the midst of early evolution, we will have grasped something important both about tubulins and the evolution of organisms that use it. In a broader sense, this single example makes it clear that continuing study of diverse eukaryotes is going to enhance our understanding in ways that focusing simply on mammals never will.

One conjecture is that Heterolobosea evolved at a time when oxygen was very limiting, and having a specialized tubulin allowed them to manage division under anoxic conditions. We know from the *Naegleria* genome that the organism has a capacity for both aerobic respiration and anaerobic metabolism (Fritz-Laylin et al., 2010). Green algae, and others separating from LECA later, could utilize mitotic machinery that depended on a higher oxygen atmosphere. Another possibility, already mentioned, is that having separate tubulins for mitosis and flagella allowed *Naegleria* to switch modules quickly. Whatever the reason, changing one’s mitotic machinery seems like a very dangerous activity, even for an investigator as bold as evolution.

The vastness of this 2200-million-year global experiment takes one’s breath away. Genomics are just beginning to reveal the complexity and subtleties of eukaryotes, as more species and groups are analyzed. The day when what’s good for the elephant is good for *Naegleria* is gone. We can expect to learn much in the coming decades. But we have certainly learned not to think of eukaryotes as a uniform group, but instead as a group whose diversity has much to teach us that our own small group, the mammals, cannot. The “far-out organism” *Naegleria* showed us multi-tubulins, as it demonstrated the capacity of centrioles to assemble *de novo*; these surprises subsequently have been extended to mammals and changed our ways of thinking. *Naegleria* has more to contribute, as do numerous other neglected protists.

In the meantime, the more we know about tubulins, the more it seems true that, paraphrasing George Orwell about the pigs in *Animal Farm*: “all tubulins are equal, but some tubulins are more equal than others” (Fulton and Simpson, 1976). Tubulins are certainly not all equal, as was generally believed in 1976, nor does each function of tubulin require a separate isotype. As we understand tubulin isotypes better, we will have a richer understanding of evolution’s playground and the diversity of eukaryotes. This is an exciting time for converting our growing data on tubulin isotypes into understanding.

## Data Availability

The original contributions presented in the study are included in the article/Supplementary Materials, further inquiries can be directed to the corresponding author.
